# The Canadian Registry for Pulmonary Fibrosis: Design and Rationale of a National Pulmonary Fibrosis Registry

**DOI:** 10.1155/2016/3562923

**Published:** 2016-04-05

**Authors:** Christopher J. Ryerson, Benjamin Tan, Charlene D. Fell, Hélène Manganas, Shane Shapera, Shikha Mittoo, Mohsen Sadatsafavi, Teresa To, Andrea Gershon, Jolene H. Fisher, Kerri A. Johannson, Nathan Hambly, Nasreen Khalil, Theodore K. Marras, Julie Morisset, Pearce G. Wilcox, Andrew J. Halayko, Mohammad Adil Khan, Martin Kolb

**Affiliations:** ^1^Department of Medicine, University of British Columbia, 1081 Burrard Street, Ward 8B, Vancouver, BC, Canada V6Z 1Y6; ^2^Centre for Heart Lung Innovation, University of British Columbia, 1081 Burrard Street, Ward 8B, Vancouver, BC, Canada V6Z 1Y6; ^3^Department of Medicine, University of Calgary, Calgary, Canada; ^4^Department of Medicine, University of Montreal, Montreal, Canada; ^5^Department of Medicine, University of Toronto, Toronto, Canada; ^6^Centre for Clinical Epidemiology and Evaluation, Vancouver Coastal Health Institute, Canada; ^7^The Hospital for Sick Children, University of Toronto, Toronto, Canada; ^8^Department of Medicine, McMaster University, Hamilton, Canada; ^9^Departments of Internal Medicine and Physiology and Pathophysiology, University of Manitoba, Winnipeg, Canada; ^10^Boehringer Ingelheim, Burlington, Canada

## Abstract

*Background*. The relative rarity and diversity of fibrotic interstitial lung disease (ILD) have made it challenging to study these diseases in single-centre cohorts. Here we describe formation of a multicentre Canadian registry that is needed to describe the outcomes of fibrotic ILD and to enable detailed healthcare utilization analyses that will be the cornerstone for future healthcare planning.* Methods*. The Canadian Registry for Pulmonary Fibrosis (CARE-PF) is a prospective cohort anticipated to consist of at least 2,800 patients with fibrotic ILD. CARE-PF will be used to (1) describe the natural history of fibrotic ILD, specifically determining the incidence and outcomes of acute exacerbations of ILD subtypes and (2) determine the impact of ILD and acute exacerbations of ILD on health services use and healthcare costs in the Canadian population. Consecutive patients with fibrotic ILD will be recruited from five Canadian ILD centres over a period of five years. Patients will be followed up as clinically indicated and will complete standardized questionnaires at each clinic visit. Prespecified outcomes and health services use will be measured based on self-report and linkage to provincial health administrative databases.* Conclusion*. CARE-PF will be among the largest prospective multicentre ILD registries in the world, providing detailed data on the natural history of fibrotic ILD and the healthcare resources used by these patients. As the largest and most comprehensive cohort of Canadian ILD patients, CARE-PF establishes a network for future clinical research and early phase clinical trials and provides a platform for translational and basic science research.

## 1. Introduction

Fibrotic interstitial lung disease (ILD) includes a heterogeneous collection of uncommon disorders that are frequently characterized by progressive decline in lung function and respiratory failure. Major fibrotic ILD subtypes include idiopathic pulmonary fibrosis (IPF), connective tissues disease-associated ILD (CTD-ILD), idiopathic nonspecific interstitial pneumonia (NSIP), and chronic hypersensitivity pneumonitis (HP) [1]. IPF is the most common fibrotic ILD, with an estimated incidence of up to 10 cases per 100,000 persons per year among people of all ages [2] and up to 94 cases per 100,000 persons per year in an elderly population [3].

Fibrotic ILDs have variable clinical features, phenotypes, outcomes, and responses to therapy. This heterogeneity complicates ILD research and necessitates large cohorts to adequately study ILD subgroups [4]. For example, a subset of IPF patients experience episodes of acute exacerbation, characterized by rapid worsening of respiratory symptoms and a short-term mortality of 40–50% [5–11]. The incidence and etiology of acute exacerbation of IPF (AE-IPF) remain unclear, predominantly because these rare events are difficult to study in single-centre studies.

Patients with fibrotic ILD have higher healthcare costs compared to matched non-ILD populations [12, 13], and these costs will predictably increase with an aging population and the use of novel and expensive medications. Previous cost analyses have relied upon health administrative databases [12, 13]; however these databases have unknown diagnostic accuracy and lack ILD-specific details that are required to identify specific determinants and patterns of health services use. These limitations indicate the need for large cohorts of well-characterized ILD patients that can be directly linked to healthcare databases, thus facilitating detailed cost-benefit analyses that apply to the Canadian setting.

Herein, we describe the Canadian Registry for Pulmonary Fibrosis (CARE-PF), the first prospective multicentre registry for ILD patients in Canada. Through CARE-PF we are (1) collecting, collating, and linking specific data components from a clinical cohort that will include at least 2,800 fibrotic ILD patients from ILD centres across Canada; (2) using these data to study the natural history of fibrotic ILDs in a Canadian population, specifically determining the incidence and outcomes of acute exacerbation within a Canadian ILD population; (3) determining the impact of ILD and acute exacerbation of ILD on health services use and healthcare costs in a Canadian population; and (4) establishing CARE-PF as a platform for future clinical and translational research on fibrotic ILDs in Canada. In the long term, the CARE-PF investigators will work with similar registries from other nations that will contribute to a better understanding of ILD in a global setting.

## 2. Methods

### 2.1. Study Overview

CARE-PF is an open-ended prospective observational cohort study of at least 2,800 patients with fibrotic ILD, with an initial term of five years. Patients will complete study questionnaires and undergo clinical measurements at baseline and follow-up visits every three to six months, as is typical clinical practice in Canada. Data will be stored in an encrypted password-protected web-based database that includes instantaneous data validation (e.g., checks for formatting, out-of-range values). Health services use data will be obtained through self-report and by linking to patient-specific data obtained from provincial and national administrative health databases.

**Figure 1:**
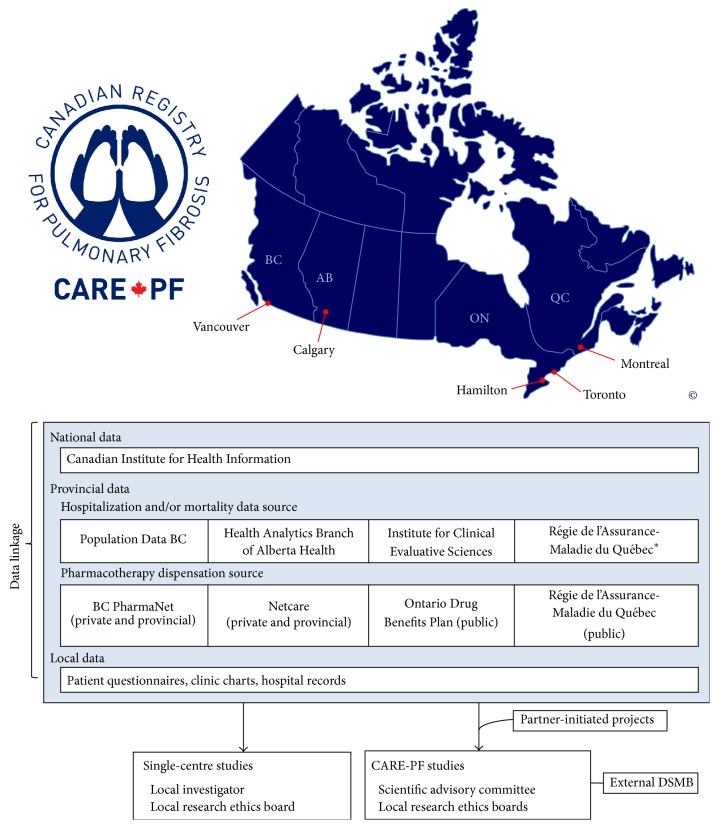
CARE-PF data sources and organizational structure. Copyright owned by the CARE-PF investigators. *∗* means Québec healthcare utilization data are available only for patients who are ≥65 years of age or on welfare.

The CARE-PF research team includes ILD clinicians, clinical and basic science researchers, epidemiologists, biostatisticians, health services researchers, and health economists. Study investigators will conduct research using study-wide data following approval from the Scientific Advisory Committee and local Research Ethics Boards. In addition, contributing investigators will have complete access to their local data, facilitating future single-centre research and quality improvement studies. Partner-initiated projects from external investigators will be proposed to the scientific advisory board and approved studies will undergo research ethics board review. An external Data Safety and Monitoring Board will oversee the registry and all proposed studies to ensure that the interests of participants are protected, including maintenance of patient privacy. The CARE-PF investigators will have ownership of the study data, and the decision to publish results will be made by the Scientific Advisory Committee, independent of the study sponsor. The CARE-PF data sources and organizational structure are summarized in Figure 1.

### 2.2. Study Participants

Consenting patients with incident or prevalent fibrotic ILD will be recruited from specialized ILD clinics at the University of British Columbia (Vancouver, BC), McMaster University (Hamilton, ON), University of Calgary (Calgary, AB), University of Toronto (Toronto, ON), and University of Montreal (Montreal, QC). Each of these institutions has dedicated ILD clinics in which patients are reviewed in a multidisciplinary setting with input from chest radiologists and lung pathologists to ensure that patients are accurately diagnosed according to established criteria [1, 14]. Our conservative recruitment estimate of 2,800 patients is based on historical volume at each ILD clinic, anticipating at least 160 prevalent cases per clinic in year 1 plus 80 incident cases in years 1 through 5. Additional sites may be added in subsequent years. All fibrotic ILD patients that meet eligibility criteria (Table 1) will be approached to determine their interest in participation. Patients with IPF comprise approximately 20% at each clinic, providing an expected IPF population of 560 patients.

### 2.3. Measurements

Patients will complete a baseline questionnaire upon enrolment into CARE-PF, including demographic features, historical data related to ILD etiology, and the patient-reported presence of common comorbidities. Pulmonary symptoms and quality of life will be measured at baseline and approximately six monthly intervals, or more frequently during periods of rapidly changing health status. Clinical measurements will be performed as indicated (Table 2).

#### 2.3.1. Diagnostic Criteria

Diagnostic criteria for fibrotic ILDs will be recorded prospectively, including connective tissue disease serology, high-resolution computed tomography (HRCT) pattern (i.e., usual interstitial pneumonia (UIP), possible UIP, and inconsistent with UIP), and histopathological pattern (i.e., UIP, probable UIP, possible UIP, nonclassifiable fibrosis, and not UIP) [14]. Diagnoses as well as HRCT and histopathological patterns will be determined based on multidisciplinary evaluation that includes a chest radiologist and lung pathologist. Patients with an unclassifiable ILD will have up to three differential diagnoses listed. The date of diagnosis will be the date on which all necessary diagnostic information was available, even if the correct diagnosis was not provided to the patient on that date. Patients with a change in diagnosis will have the date of the new diagnosis recorded in a similar manner (e.g., an IPF patient who subsequently develops rheumatoid arthritis with a change in diagnosis to rheumatoid arthritis associated ILD).

#### 2.3.2. Baseline Clinical Data

Demographics and baseline clinical data will be recorded using a standardized questionnaire that includes race, smoking history, family history, medication exposures (i.e., medications that can cause pulmonary fibrosis), occupational exposures, environmental exposures, and the presence of comorbid diseases. The presence of comorbidities will be assessed using patient-reported questionnaires, including the Charlson Comorbidity Index [9, 10].

#### 2.3.3. Patient-Reported Outcomes

Dyspnea will be assessed using the University of California San Diego Shortness of Breath Questionnaire (UCSD SOBQ) [15, 16]. The UCSD SOBQ is a 24-item questionnaire that asks patients to rate the severity of breathlessness they would experience when conducting common activities of varying intensity. The UCSD SOBQ has been used in previous studies of ILD, including randomized trials of pharmacotherapies in IPF. Self-assessed cough severity will be measured using a 10 cm visual analog scale. Additional symptoms will be recorded based on patient self-report (e.g., extrapulmonary manifestations of CTD-ILD).

Quality of life will be measured using the St. George's Respiratory Questionnaire (SGRQ) and the European Quality of Life 5 Dimensions questionnaire (EQ-5D). The SGRQ is the most commonly used and best-studied quality of life questionnaire in ILD [17] and can also be used to calculate disease-specific quality of life for IPF (SGRQ-i) [18]. The SGRQ is a 50-item questionnaire that has a minimum clinically important difference of 5–8 points in IPF [19]. The EQ-5D is a 5-item quality of life questionnaire with an additional visual analog scale [20–22]. The EQ-5D has also been used in IPF clinical trials and has established Canadian population norms that can assign values for health states to allow calculation of quality-adjusted life years (QALYs) [23].

#### 2.3.4. Physiological Measurements

Spirometry, lung volumes, and diffusing capacity of the lung for carbon monoxide (DLCO) will be measured using standard techniques when clinically indicated [24–26]. Each province has an accreditation program that ensures appropriate quality control. The frequency of testing will be based on clinical indication. Height and weight will be recorded at the time of testing to permit recalculation of percent-predicted values using absolute pulmonary function measurements and consistent reference equations.

Six-minute walk distance (6 MWD) and exertional oxygen saturation will be measured according to standard techniques when clinically indicated [27, 28]. The 6 MWD will be reported in metres and as a percent-predicted value, based on sex, age, height, and weight as previously described [29].

#### 2.3.5. Additional Measurements

Echocardiogram, right heart catheterization, and bronchoscopy findings will be recorded using a standard template when performed. The decision to perform these investigations will be made by the treating physician based on clinical indication.

### 2.4. ILD Treatments

Study participants will be managed according to standard clinical practice. All pharmacologic and nonpharmacologic interventions will be initiated and terminated according to clinical judgment and supported by guideline recommendations where applicable. ILD treatments will be recorded prospectively using a standardized format, including both pharmacologic and nonpharmacologic therapies.

### 2.5. Outcome Measurements

#### 2.5.1. ILD Progression

The rate of ILD progression will be determined based on repeated measurements of forced vital capacity (FVC) and DLCO. Most patients will have pulmonary function measurements obtained at six-month intervals; however some patients may have more or less frequent follow-up depending on clinical need. The Composite Physiologic Index will be used as an additional estimate of ILD severity, based on concurrent measurements of FVC, FEV_1_, and DLCO [30].

#### 2.5.2. Hospitalizations and Acute Exacerbation of ILD

Study investigators will record dates of hospitalization and the reason for hospitalization. Acute exacerbation of IPF will be defined as unexplained worsening of dyspnea and new bilateral ground glass or consolidative change over the preceding 30 days in a patient with a diagnosis of IPF [5]. A central adjudication committee will review clinical, laboratory, and imaging data to identify “confirmed” acute exacerbations, defined as events that have had infection excluded by bronchoscopic or endotracheal aspirate sampling. “Suspected” acute exacerbation of IPF will include events meeting all criteria for acute exacerbation of IPF, but without bronchoscopy or endotracheal aspiration [31]. Acute exacerbation of other ILD subtypes will be assessed and recorded with a similar definition.

#### 2.5.3. Health Services Use

Patient-level data will be linked to respective provincial health administrative databases to capture inpatient, emergency department visits, and outpatient health services use. Consent to use provincial personal health numbers (PHNs) to link clinical and provincial data will be sought from all patients. Provincial data available for linkage are listed in Figure 1. All-cause and cause-specific health services use will be determined. Crude, age-, and sex-specific rates of health services use will be calculated.

#### 2.5.4. Healthcare Costs

Healthcare costs will be determined from province-specific data sources as outlined above, as well as provincial drug dispensation databases as listed in Figure 1. These data will be cross-referenced with clinical records to ensure completeness. For estimating the costs of hospitalizations, we will use the case mix methodology, by multiplying the resource intensity weight available in the data, with the provincial cost per weighted case [32]. The lowest available price will be used for all medications from provincial drug master files (i.e., generic brand if available). Costs of outpatient services use (e.g., physician visits) will be determined by cross-referencing the service codes with fee-for-service information within each province.

#### 2.5.5. Death and Lung Transplantation

Dates of death and lung transplantation will be based on provincial data sources and review of the clinical record. Cause of death will be determined based on review of the death certificate and clinical records.

### 2.6. Statistical Analysis

The rate of change in FVC and DLCO will be determined using mixed effects models to account for nonstandardized follow-up schedules and patient dropout. Additional analyses will consider change in FVC as a dichotomous outcome (e.g., 5% and 10% absolute decline over 6–12 months). Subgroup analyses will be conducted for specific ILD subtypes and to compare the rate of physiological progression among subgroups. These analyses will adjust for potential confounders (e.g., age, sex, and baseline ILD severity), as well as additional variables identified on screening analysis.

The annual incidence of acute exacerbation of IPF will be determined within a population of patients with IPF. Subgroup analyses will be performed to determine the 1-year incidence of AE-IPF in patients with a new (incident) diagnosis of IPF, the annual incidence in subsequent years, and the incidence in patients with varying fibrosis severity. Similar analyses will be conducted in other fibrotic ILD subtypes. Outcomes following acute exacerbation will be reported as 30-day mortality, in-hospital mortality, median survival after exacerbation, and postsurvival quality of life. Multivariate analyses will be used to identify risk factors for acute exacerbation, as well as risk factors for mortality in patients experiencing exacerbation. Patients with multiple exacerbations will contribute only the first event to these analyses.

Healthcare services use and healthcare costs will be determined for ILD patients and for individual acute exacerbation events as described above. Healthcare services use and healthcare costs will be compared to a general non-ILD population. Disease controls will include patients with chronic obstructive pulmonary disease (COPD) and acute exacerbations of COPD. Four matched controls for each case will be identified from provincial databases, matching for age, sex, and geographic area of residence. Additional analyses will evaluate the change in health services use and healthcare costs in ILD patients over time and with changing lung function (e.g., stratified by mild, moderate, and severe ILD).

## 3. Discussion

Previous studies have provided valuable data on the epidemiology, clinical features, and outcomes of fibrotic ILD; however these studies have methodological limitations. There is therefore an unmet need for a large well-phenotyped multicentre cohort that will allow detailed study of these populations in a Canadian healthcare environment.

The primary goal of CARE-PF is to improve our understanding of the natural history of fibrotic ILD subtypes and specifically to determine the incidence, risk factors, clinical features, and prognosis of acute exacerbation of IPF and other ILDs. Previous studies report an annual AE-IPF incidence of 5–15% per year [33]. Assuming 30% annual attrition, this incidence suggests that we would observe between 63 and 189 AE-IPF events during this 5-year study that includes 560 patients with IPF (2,800 total ILD patients). Our anticipated sample size will therefore allow detailed characterization of these important but uncommon outcomes. CARE-PF will include linkage to provincial health databases that will identify hospital admissions that are not captured in outpatient records at each ILD clinic. This will help identify events that were not reported to study investigators, thus increasing the sensitivity for identification of acute exacerbations and providing more accurate estimates of incidence and outcome.

A second goal of CARE-PF is to determine the impact of ILD and acute exacerbation of ILD on health services use and healthcare costs in a Canadian population. A recent study showed that healthcare costs are almost twofold higher in IPF compared to non-IPF controls [13]; however these findings are based upon potentially inaccurate ICD-10 coding and Medicare data that only applies to elderly patients in the United States. The single-payer structure of the centralized Canadian healthcare system will allow CARE-PF to address some of these limitations by linking well-characterized ILD patients to patient-specific provincial data that includes health services use and healthcare costs. CARE-PF is therefore ideally situated to determine the cost implications of fibrotic ILD, thus providing important data that can be used by stakeholders (patients, governments, industry, and healthcare leaders) to inform decisions on treatment, legislature, and research directions.

We chose to initially focus CARE-PF on five major ILD centres for several reasons. First, inclusion of only major ILD centres allows for rapid recruitment of well-characterized ILD patients. Furthermore, the relatively small number of centres during this initial phase will simplify quality control and troubleshooting, ensuring high-quality data. Third, the existing infrastructure at these experienced centres will ease the administrative burden and cost of initiating this study. Our focus on major ILD centres introduces a known sample bias; however this will be partly attenuated by the broad referral base of these clinics that receive referrals from general practitioners and community respirologists. CARE-PF is not a cross-sectional sampling of the Canadian population and is therefore unable to inform the incidence and prevalence of fibrotic ILDs. These limitations could be addressed by future expansion of CARE-PF to include additional academic ILD centres and community clinics.

## 4. Conclusions

CARE-PF is the first study in Canada to provide robust epidemiological data on ILD. CARE-PF will also serve as a platform to support future translational and basic science research, with plans to establish biobanks that can be used to catalogue novel biomarkers to enable patient stratification, predict acute exacerbation risk, and monitor response to therapy. Finally, CARE-PF is designed to parallel other global initiatives, thus facilitating establishment of a global collaborative network that is essential for further advancement of pulmonary fibrosis research [4].

## Figures and Tables

**Table 1:** Eligibility criteria.

Inclusion criteria	(i) Fibrotic ILD of any subtype (ii) At least 18 years of age (iii) Able to provide informed consent (iv) Able to complete study questionnaires in English or French

Exclusion criteria	(i) None

**Table 2:** Schedule of study measurements.

Measurements	Baseline	Follow-up^**∗**^
Diagnostic criteria	X	+/−
Connective tissue disease serology		
HRCT: UIP pattern and favoured diagnosis		
Surgical lung biopsy: histological pattern		

Baseline clinical data	X	+/−
Demographics (e.g., age, sex, race)		
Smoking history		
Family history		
Medication exposures		
Occupational exposures		
Environmental exposures		
Comorbidities		

Symptoms	X	X
Dyspnea (UCSD SOBQ)		
Cough (visual analogue scale)		
Additional symptoms		

Quality of life	X	X
St. George's Respiratory Questionnaire		
European Quality of Life 5 Dimensions questionnaire		

Pulmonary function tests	X	X

6-minute walk tests	X	X

Additional measurements^**∗∗**^	+/−	+/−

^*∗*^Follow-up data will be obtained when clinically indicated. Changes to diagnosis and baseline data will be recorded if applicable (e.g., a change in diagnosis and new comorbidity).

^*∗∗*^Additional measurements will be recorded when performed (e.g., echocardiogram, right heart catheterization, and bronchoscopy).

HRCT, high-resolution computed tomography; UIP, usual interstitial pneumonia; UCSD SOBQ, University of California San Diego Shortness of Breath Questionnaire.
